# 3D-modeling of the spine using EOS imaging system: Inter-reader reproducibility and reliability

**DOI:** 10.1371/journal.pone.0171258

**Published:** 2017-02-02

**Authors:** Johannes Rehm, Thomas Germann, Michael Akbar, Wojciech Pepke, Hans-Ulrich Kauczor, Marc-André Weber, Daniel Spira

**Affiliations:** 1 Diagnostic and Interventional Radiology, University Hospital, Heidelberg, Germany; 2 Clinic for Orthopedics and Trauma Surgery, University Hospital, Heidelberg, Germany; Seoul National University College of Medicine, REPUBLIC OF KOREA

## Abstract

**Objectives:**

To retrospectively assess the interreader reproducibility and reliability of EOS 3D full spine reconstructions in patients with adolescent idiopathic scoliosis (AIS).

**Methods:**

73 patients with mean age of 17 years and a moderate AIS (median Cobb Angle 18.2°) obtained low-dose standing biplanar radiographs with EOS. Two independent readers performed “full spine” 3D reconstructions of the spine with the “full-spine” method adjusting the bone contour of every thoracic and lumbar vertebra (Th1-L5). Interreader reproducibility was assessed regarding rotation of every single vertebra in the coronal (i.e. frontal), sagittal (i.e. lateral), and axial plane, T1/T12 kyphosis, T4/T12 kyphosis, L1/L5 lordosis, L1/S1 lordosis and pelvic parameters. Radiation exposure, scan-time and 3D reconstruction time were recorded.

**Results:**

Interclass correlation (ICC) ranged between 0.83 and 0.98 for frontal vertebral rotation, between 0.94 and 0.99 for lateral vertebral rotation and between 0.51 and 0.88 for axial vertebral rotation. ICC was 0.92 for T1/T12 kyphosis, 0.95 for T4/T12 kyphosis, 0.90 for L1/L5 lordosis, 0.85 for L1/S1 lordosis, 0.97 for pelvic incidence, 0.96 for sacral slope, 0.98 for sagittal pelvic tilt and 0.94 for lateral pelvic tilt. The mean time for reconstruction was 14.9 minutes (reader 1: 14.6 minutes, reader 2: 15.2 minutes, p<0.0001). The mean total absorbed dose was 593.4μGy ±212.3 per patient.

**Conclusion:**

EOS “full spine” 3D angle measurement of vertebral rotation proved to be reliable and was performed in an acceptable reconstruction time. Interreader reproducibility of axial rotation was limited to some degree in the upper and middle thoracic spine due the obtuse angulation of the pedicles and the processi spinosi in the frontal view somewhat complicating their delineation.

## Introduction

The advantages of three-dimensionally analyzing and quantifying adolescent idiopathic scoliosis (AIS) for follow-up and therapy planning are well known [1–2]. Apart from the clinical examination, regular roentgenologic monitoring with two plane radiographs are the gold standard and are fundamental to detect progress and estimate the prognosis. In addition X-rays serve as pre- and postoperative assessment tools in case of spondylodesis. Although modern techniques have been able to reduce radiation exposure, ionizing radiation of conventional plane radiographs was extrapolated to increase the lifetime risk of developing breast and thyroid cancer by 1–2%, especially in young patients with AIS [3–4]. Two-dimensional images are limited in their ability to assess vertebral rotation and pelvic parameters, which is why an additional CT is sometimes needed to increase accuracy of measurements. Besides radiation exposure the main disadvantage of the CT is the supine positioning of the patient during the examination, which can lead to considerable differences in vertebral rotation and extent of scoliosis when compared to upright posture. The new EOS-technology (EOS imaging, Paris, France) based on a low-dose X-ray system allows 3D modeling of the spine based on 2-dimensional X-rays acquired in an upright position providing information about scoliosis and sagittal balance. In addition, it provides information about pelvic parameters. The core of the EOS system is a multiwire proportional chamber with two independent two independent X-ray tubes producing a 45-cm-wide X-ray beam and image acquisition plates complete the system [5]. The X-ray system surrounds the chamber the patient stands within and scans the patient longitudinally in a weight-bearing position over a preset area. The sterEOS (EOS imaging, Paris, France) software enables 3D modeling of the bone envelope based on anatomic references defined by the reader and providing specific clinical parameters [6–7]. Although EOS is sometimes equated with CT due to its ability to provide 3D reconstructions, it does not provide information on soft tissues. In recent studies the validity of EOS imaging in a preoperative and postoperative setting as well as in the follow up of patients with AIS has been investigated. The EOS images were found to be superior or equivalent to conventional radiographs in terms of global image quality and structure visibility with up to nine times lower radiation [8–10].

As AIS is a complex multidirectional spinal deformity, the analysis of the vertebral rotation in all three dimensions helps to characterize and understand the true shape of the deformity during follow-up and for therapy planning. In addition it can quantify the surgical outcome. To our knowledge there is no differentiated analysis on reliability of the three-dimensional rotation measurements with EOS of all thoracolumbar vertebral bodies so far. Former studies only focused on the rotation of the apical vertebra (AVR) [7,9,11]. We therefore set out to assess the interreader reproducibility and reliability of the EOS full-spine method in patients with AIS referring to the rotation of every single vertebra (T1-L5) in the coronal (i.e. frontal), sagittal (i.e. lateral), and axial plane.

## Materials and methods

### Patients

This retrospective study was approved by our Ethics Committee (vote S-0627/2015). 73 consecutive patients (31 men, 42 woman) with AIS underwent an examination of the whole spine with the EOS imaging system between July 2015 and November 2015. Mean age was 17 years (range, 9–58 years) and mean Cobb angle was 18.2° (range, 9.8°-49.9°).

Exclusion criteria were lumbosacral transitional vertebrae (n = 4) and vertebral deformity, i.e. butterfly vertebra (n = 1) and hemivertebra (n = 1), as well as motion artifacts (n = 2). According to these criteria 8 patients dropped out initially. Furthermore patients with metallic implants from a previous spine surgery (e.g. spondylodesis, vertebral body or disc replacement) were not included in this study. Low-dose biplanar (AP and lateral) X-rays were obtained with the EOS system in a weight bearing position with arms folded at 45° to reduce superimposition on the spine as previously described [8]. Patients were asked to hold their breath during the examination. The images included the last cervical vertebra (C7), the pelvis and both femoral heads.

### Data collection and 3D reconstructions

Demographic data included age and gender. Scan-time, radiation exposure, kilovolts and milliamperes were recorded in all EOS biplanar X-rays. Preset exposure parameters were 90kV and 200mA for the anteroposterior (AP) view and 105kV and 250mA for the lateral view. The 3D reconstructions were performed using the “full spine” protocol with the dedicated sterEOS software by two independent readers (radiologists with 2 and 3 years work experience, respectively). Each reader attended a detailed training session with an EOS representative.

**Fig 1:**
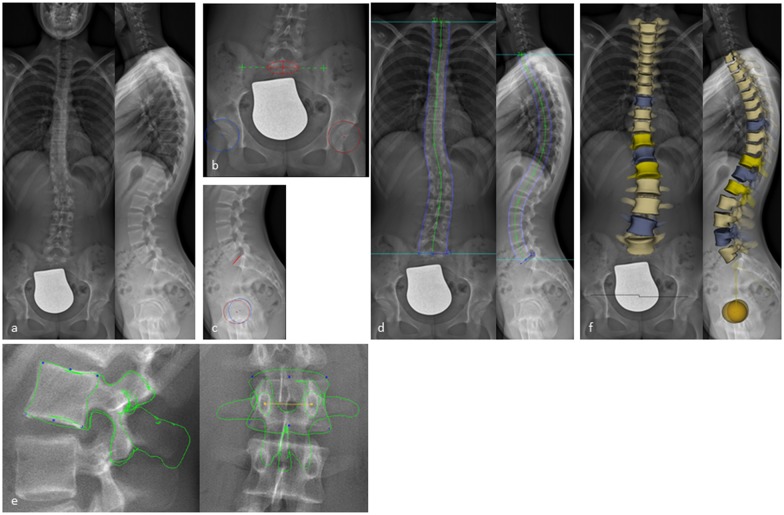
3D reconstruction from biplanar X-rays using sterEOS. (a) Biplanar X-rays performed with EOS in a standing position. (b,c) Digitalization of primary anatomical landmarks on the pelvis defining the position of the sacrum (red circle with a cross in the a.p. and red line in the lateral view) and the iliosacral joints (green crosses) as well as on both acetabuli (red and blue circles with a cross in the middle). (d) Shaping the spine from T1 to L5 using green control points. (e) Shaping every vertebral body (T1-L5) by identifying anatomical landmarks using blue and yellow control points on the vertebral bodies resulting in a 3D full spine model (f).

To obtain 3D reconstructions, primary anatomical landmarks were marked on the pelvis (two spheres on the acetabula and a segment on the sacral endplate). Then every single vertebral body (T1-L5) was traced by identifying anatomical landmarks using control points on the vertebral bodies (endplates, pedicles, processi transversi/spinosi and posterior arches) resulting in a 3D full spine model, as previously described by Humbert at al. [7]. The software allows adjustments of luminosity and contrast which helped to identify the anatomical landmarks named above (Fig 1). The following measurements were calculated from the 3D reconstruction and provided in each patient`s report: frontal, lateral and axial rotation of every thoracic and lumbar vertebra (T1-L5), T1/T12 kyphosis, T4/T12 kyphosis, L1/L5 lordosis, L1/S1 lordosis, pelvic incidence, sacral slope, sagittal pelvic tilt and lateral pelvic tilt. The time required by each reader to perform the 3D reconstruction was recorded.

### Statistical analysis

The statistical analysis was performed by the lead statistician of a biometric institute (Statistical and biometrical solutions, Zweibrücken, Germany) using the statistical software SAS 9.3 (SAS Institute Inc., North Carolina). All data are reported as arithmetic mean ± S.D. or range, as appropriate.

Demographic information as well as measurement characteristics were analyzed descriptively. Qualitative variables were analyzed by calculating frequencies and percentages of observed levels. Reconstruction data as well as absolute values of reconstruction data were summarized separately for all parameters (RT1-L5 and pelvic parameters), all views (frontal, lateral and axial) and both raters. Agreement among both raters was assessed graphically by Bland-Altman Plots and estimation of the 95% limits of agreement. In a Bland-Altman plot the differences in ratings between raters were presented as a function of their averages and 95% limits of agreement were plotted (Bland Altmann, 1999). To quantify the inter-reader reliability the intraclass correlation coefficient (ICC) was used. Raters were considered as a random sample of observers from a larger population of potential observers. According to Shrout and Fleiss, a two-way random-effect model with subject and rater as random effects was applied for estimation of ICC and 95% confidence intervals [12]. In addition, a summarizing plot was performed displaying ICC and 95% confidence interval for all parameters. An ICC greater than 0.91, between 0.71 and 0.91, between 0.51 and 0.70, or less than 0.51 was considered to represent, respectively, very good, good, moderate or poor agreement, according to former studies [9,13]. Reconstruction times of both readers were analyzed descriptively and compared by using paired t-test or Wilcoxon paired signed rank test if the requirements of the t-test were not fulfilled. P-values were presented as two-sided p-values and level of significance was set to 5%.

## Results

### Radiation exposure, scan-time and reconstruction data

The mean effective voltage was 87.6kV ±3.34 for AP and 104kV ±1.43 for lateral view. The mean effective amperage was 206.2mA ±21.5 for the AP and 240.6mA ±38.45 for the lateral view resulting in a mean absorbed dose of 253.2μGy ±97.5 for the AP and 339.4μGy ±119.47 for the lateral view (mean of total absorbed dose 593.4μGy ±212.3).

The mean scan-time was 9.5 seconds ±1.7. Reconstruction time differed significantly between both readers (p<0.0001). The mean reconstruction time of reader 1 was 14.6 minutes ±1.38 whereas the mean reconstruction time of reader 2 was 15.2 minutes ±1.52.

### Inter-reader reproducibility and reliability

Mean absolute difference of vertebral rotation between reader 1 and reader 2 ranged between 1.0° and 2.2° for frontal view, between 0.9° and 3.4° for lateral view and between 1.9° and 3.1° for axial view (Tables 1–3).

**Table 1:** Means of frontal vertebral rotation.

		Reader 1	Reader 2	absolute difference
**R T1**	Mean ± SD	3.7 ± 3.50	3.7 ± 3.19	2.0 ± 1.92
**R T2**	Mean ± SD	5.0 ± 4.81	4.7 ± 4.25	2.2 ± 2.66
**R T3**	Mean ± SD	5.1 ± 5.28	4.7 ± 4.47	2.0 ± 2.69
**R T4**	Mean ± SD	6.2 ± 5.83	5.9 ± 5.52	1.7 ± 1.89
**R T5**	Mean ± SD	6.7 ± 6.53	6.8 ± 6.12	1.3 ± 1.26
**R T6**	Mean ± SD	6.4 ± 6.70	6.2 ± 6.12	1.4 ± 1.45
**R T7**	Mean ± SD	5.0 ± 5.52	5.0 ± 5.55	1.1 ± 1.06
**R T8**	Mean ± SD	4.2 ± 5.00	4.4 ± 4.77	1.0 ± 0.82
**R T 9**	Mean ± SD	4.1 ± 4.70	4.4 ± 4.50	1.1 ± 0.93
**R T10**	Mean ± SD	5.8 ± 5.37	5.6 ± 5.57	1.3 ± 1.24
**R T11**	Mean ± SD	6.7 ± 6.27	7.2 ± 6.28	1.5 ± 1.42
**R T12**	Mean ± SD	6.5 ± 6.23	7.0 ± 6.54	1.6 ± 1.54
**R L1**	Mean ± SD	5.8 ± 5.27	5.9 ± 5.35	1.7 ± 1.47
**R L2**	Mean ± SD	5.1 ± 5.05	5.5 ± 4.81	1.5 ± 1.35
**R L3**	Mean ± SD	5.6 ± 5.53	6.2 ± 5.51	1.4 ± 1.30
**R L4**	Mean ± SD	6.0 ± 5.09	6.2 ± 4.81	1.0 ± 0.76
**R L5**	Mean ± SD	4.1 ± 2.99	4.1 ± 3.52	1.4 ± 1.42

The mean ± SD of the vertebral rotation (R) in the frontal view was calculated on every level (RT1- RL5) for each reader. In addition the absolute difference was determined.

**Table 2:** Lateral vertebral rotation.

		Reader 1	Reader 2	absolute difference
**R T1**	Mean ± SD	18.9 ± 9.14	21.6 ± 9.08	3.4 ± 2.46
**R T2**	Mean ± SD	19.9 ± 9.53	21.1 ± 9.48	2.5 ± 2.06
**R T3**	Mean ± SD	17.7 ± 9.52	18.4 ± 9.36	1.8 ± 1.70
**R T4**	Mean ± SD	13.7 ± 9.04	14.5 ± 9.06	1.7 ± 1.22
**R T5**	Mean ± SD	10.7 ± 7.82	11.1 ± 7.86	1.4 ± 0.96
**R T6**	Mean ± SD	7.4 ± 6.43	7.7 ± 6.36	1.3 ± 1.17
**R T7**	Mean ± SD	5.9 ± 4.58	5.9 ± 4.65	1.4 ± 1.16
**R T8**	Mean ± SD	6.4 ± 4.68	6.3 ± 4.72	1.2 ± 0.89
**R T9**	Mean ± SD	9.3 ± 5.07	9.1 ± 5.06	0.9 ± 0.73
**R T10**	Mean ± SD	12.7 ± 6.85	12.4 ± 6.45	1.2 ± 1.14
**R T11**	Mean ± SD	15.6 ± 7.66	15.5 ± 7.45	1.4 ± 1.18
**R T12**	Mean ± SD	18.6 ± 7.96	18.3 ± 8.23	1.3 ± 1.00
**R L1**	Mean ± SD	19.0 ± 7.12	19.1 ± 7.55	1.1 ± 0.97
**R L2**	Mean ± SD	16.7 ± 6.53	16.8 ± 6.72	1.1 ± 0.94
**R L3**	Mean ± SD	10.5 ± 6.78	10.5 ± 6.53	1.3 ± 1.12
**R L4**	Mean ± SD	5.9 ± 4.29	6.2 ± 4.71	1.6 ± 1.52
**R L5**	Mean ± SD	17.5 ± 8.70	19.4 ± 8.48	2.3 ± 1.42

The mean ± SD of the vertebral rotation (R) in the frontal view was calculated on every level (RT1- RL5) for each reader. In addition the absolute difference was determined.

**Table 3:** Axial vertebral rotation.

		Reader 1	Reader 2	absolute difference
**R T1**	Mean ± SD	4.3 ± 4.01	4.0 ± 3.34	2.1 ± 3.48
**R T2**	Mean ± SD	4.4 ± 3.81	4.1 ± 3.49	2.6 ± 3.90
**R T3**	Mean ± SD	4.5 ± 3.67	4.2 ± 3.88	2.8 ± 3.39
**R T4**	Mean ± SD	4.3 ± 3.31	4.3 ± 4.05	2.8 ± 3.37
**R T5**	Mean ± SD	4.3 ± 3.01	3.8 ± 3.15	2.9 ± 2.97
**R T6**	Mean ± SD	3.5 ± 3.48	3.9 ± 3.31	3.2 ± 3.74
**R T7**	Mean ± SD	3.8 ± 3.18	4.2 ± 3.82	3.1 ± 3.41
**R T8**	Mean ± SD	4.3 ± 4.32	4.6 ± 3.91	2.9 ± 2.93
**R T9**	Mean ± SD	4.8 ± 4.31	4.7 ± 4.05	2.6 ± 2.94
**R T10**	Mean ± SD	5.0 ± 4.32	4.3 ± 3.55	2.3 ± 2.95
**R T11**	Mean ± SD	5.5 ± 4.50	4.9 ± 4.07	3.0 ± 3.10
**R T12**	Mean ± SD	5.7 ± 5.08	5.3 ± 4.72	2.9 ± 3.79
**R L1**	Mean ± SD	5.7 ± 4.89	5.6 ± 5.24	2.4 ± 3.00
**R L2**	Mean ± SD	5.0 ± 4.10	4.7 ± 4.01	2.1 ± 3.05
**R L3**	Mean ± SD	4.6 ± 3.95	4.3 ± 3.37	2.3 ± 3.13
**R L4**	Mean ± SD	3.9 ± 3.41	3.8 ± 2.76	1.9 ± 3.15
**R L5**	Mean ± SD	3.9 ± 3.05	3.8 ± 2.96	2.3 ± 2.48

The mean ± SD of the vertebral rotation (R) in the frontal view was calculated on every level (RT1- RL5) for each reader. In addition the absolute difference was determined.

Mean absolute difference for kyphosis and lordosis parameters (T1/T12 kyphosis, T4/T12 kyphosis, L1/L5 lordosis and L1/L5 lordosis) ranged between 3.0° and 4.1°. Mean absolute difference of values for pelvic parameters (pelvic incidence, sacral slope and sagittal pelvic tilt) ranged between 1.0° and 3.1° and was 0.9mm for lateral pelvic tilt respectively (Table 4).

**Table 4:** Kyphosis/lordosis and pelvic parameter.

		Reader 1	Reader 2	absolute difference
**T1/T12 kyphosis**	Mean ± SD	37.0 ± 13.65	39.1 ± 14.21	4.1 ± 4.33
**T4/T12 kyphosis**	Mean ± SD	33.0 ± 15.02	33.6 ± 15.39	3.0 ± 3.94
**L1/L5 lordosis**	Mean ± SD	42.4 ± 12.62	44.8 ± 13.44	3.8 ± 4.95
**L1/S1 lordosis**	Mean ± SD	53.7 ± 13.81	55.7 ± 14.63	4.1 ± 6.82
**Pelvic incidence**	Mean ± SD	44.0 ± 11.48	46.3 ± 11.14	3.1 ± 2.13
**Sacral slope**	Mean ± SD	36.0 ± 9.16	38.0 ± 8.86	2.7 ± 1.76
**Sagittal pelvic tilt**	Mean ± SD	8.5 ± 7.28	8.7 ± 7.20	1.0 ± 1.34
**Lateral pelvic tilt**	Mean ± SD	4.7 ± 3.25	4.7 ± 3.53	0.9 ± 0.73

The mean ± SD of the measured values for kyphosis and lordosis for each reader were assessed. In addition the absolute difference was determined.

**Fig 2:**
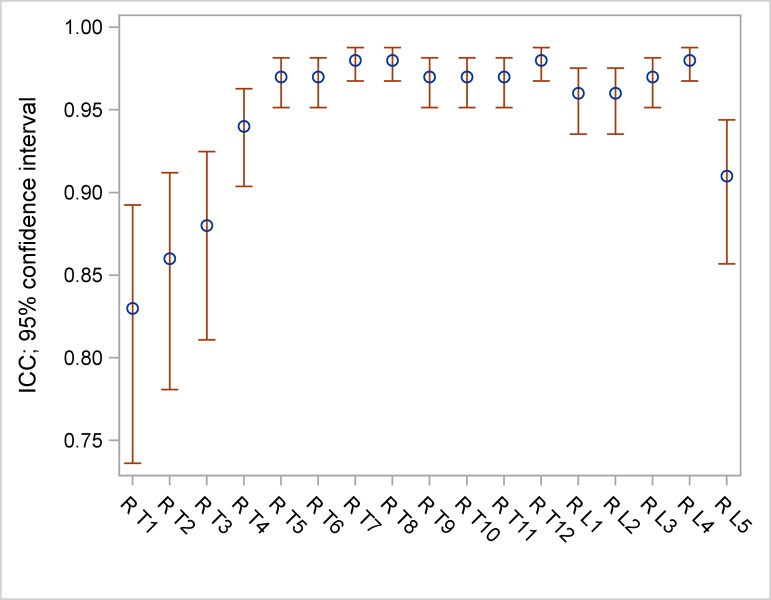
ICC and 95% confidence intervals (CI) of frontal measurements.

**Fig 3:**
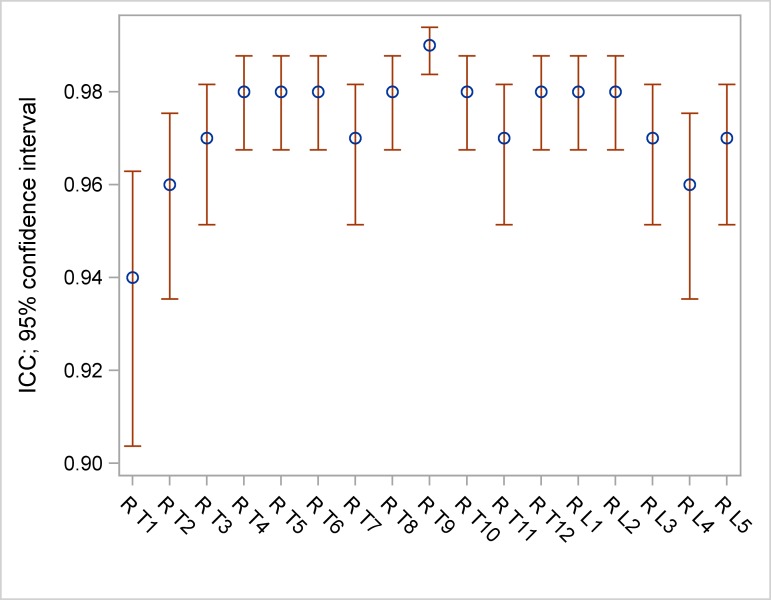
ICC and 95% confidence intervals (CI) of lateral measurements.

**Fig 4:**
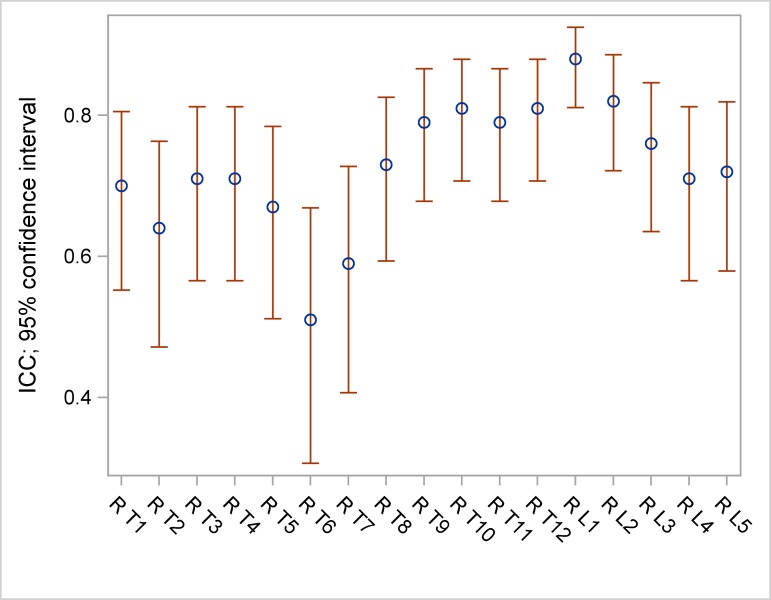
ICC and 95% confidence intervals (CI) of axial measurements.

ICC ranged between 0.83 and 0.98 for frontal vertebral rotation (Table 5, Fig 2), between 0.94 and 0.99 for lateral vertebral rotation (Table 5, Fig 3) and between 0.51 and 0.88 for axial vertebral rotation (Table 5, Fig 4).

**Table 5:** ICC and 95% confidence intervals (CI) of frontal, lateral and axial measurements.

Frontal	ICC	95%CI	Lateral	ICC	95%CI	Axial	ICC	95% CI
**R T1**	0.83	[0.74; 0.89]	**R T1**	0.94	[0.90; 0.96]	**R T1**	0.70	[0.55; 0.81]
**R T2**	0.86	[0.78; 0.91]	**R T2**	0.96	[0.94; 0.98]	**R T2**	0.64	[0.47; 0.76]
**R T3**	0.88	[0.81; 0.92]	**R T3**	0.97	[0.95; 0.98]	**R T3**	0.71	[0.57; 0.81]
**R T4**	0.94	[0.90; 0.96]	**R T4**	0.98	[0.97; 0.99]	**R T4**	0.71	[0.57; 0.81]
**R T5**	0.97	[0.95; 0.98]	**R T5**	0.98	[0.97; 0.99]	**R T5**	0.67	[0.51; 0.78]
**R T6**	0.97	[0.95; 0.98]	**R T6**	0.98	[0.97; 0.99]	**R T6**	0.51	[0.31; 0.67]
**R T7**	0.98	[0.97; 0.99]	**R T7**	0.97	[0.95; 0.98]	**R T7**	0.59	[0.41; 0.73]
**R T8**	0.98	[0.97; 0.99]	**R T8**	0.98	[0.9; 0.99]	**R T8**	0.73	[0.59; 0.83]
**R T9**	0.97	[0.95; 0.98]	**R T9**	0.99	[0.98; 0.99]	**R T9**	0.79	[0.68; 0.87]
**R T10**	0.97	[0.95; 0.98]	**R T10**	0.98	[0.97; 0.99]	**R T10**	0.81	[0.71; 0.88]
**R T11**	0.97	[0.95; 0.98]	**R T11**	0.97	[0.95; 0.98]	**R T11**	0.79	[0.68; 0.87]
**R T12**	0.98	[0.97; 0.99]	**R T12**	0.98	[0.97; 0.99]	**R T12**	0.81	[0.7; 0.88]
**R L1**	0.96	[0.94; 0.98]	**R L1**	0.98	[0.97; 0.99]	**R L1**	0.88	[0.81; 0.92]
**R L2**	0.96	[0.94; 0.98]	**R L2**	0.98	[0.97; 0.99]	**R L2**	0.82	[0.72; 0.89]
**R L3**	0.97	[0.95; 0.98]	**R L3**	0.97	[0.95; 0.98]	**R L3**	0.76	[0.64; 0.85]
**R L4**	0.98	[0.97; 0.99]	**R L4**	0.96	[0.94; 0.98]	**R L4**	0.71	[0.57; 0.81]
**R L5**	0.91	[0.86; 0.94]	**R L5**	0.97	[0.95; 0.98]	**R L5**	0.72	[0.58; 0.82]

The ICC as well as the 95% confidence intervals were assessed for the vertebral rotation measurements on the frontal, lateral and axial views.

**Fig 5:**
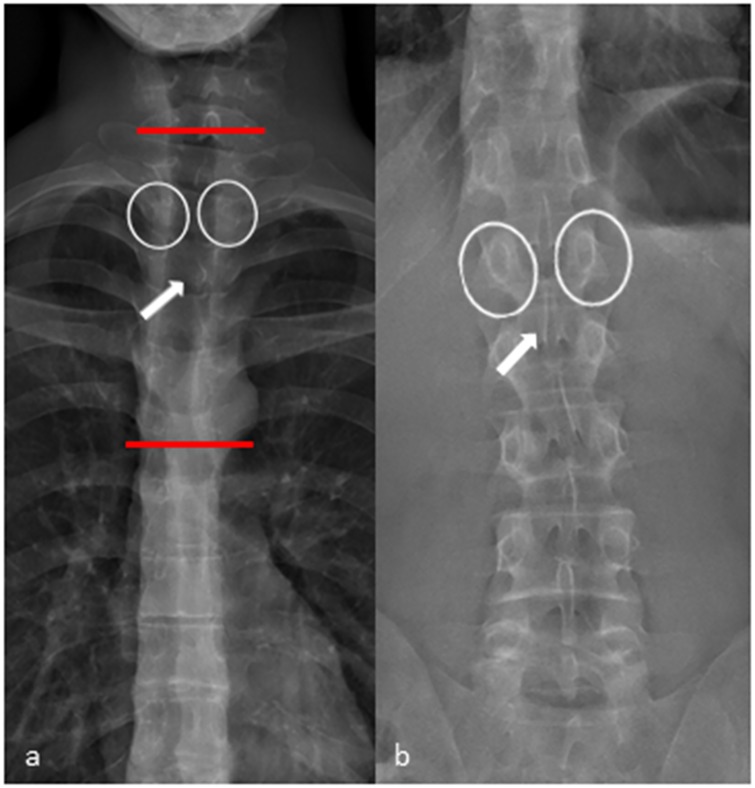
Frontal view X-ray performed with EOS. (a) Frontal view of the thoracic spine. The pedicles (white circles) and the shape of the processus spinosus (white arrow) are difficult to identify in the upper and middle thoracic spine (area in between the red lines). (b) In the lower thoracic spine and in the lumbar spine identifying the pedicles (white circles) and the shape of the proccessus spinosus (white arrow) is straight forward.

In the upper and middle thoracic spine (T1-T7) the assessment of the axial vertebral rotation had some limitations resulting in a moderate ICC. The axial rotation is mainly dependent on the adjustment of the pedicles and the posterior arch in the frontal view. Identifying these structures can be difficult in the upper and middle thoracic spine because of their lower diameter and the obtuse angle of the pedicles and the posterior arch in comparison to the lumbar spine (Fig 5).

**Fig 6:**
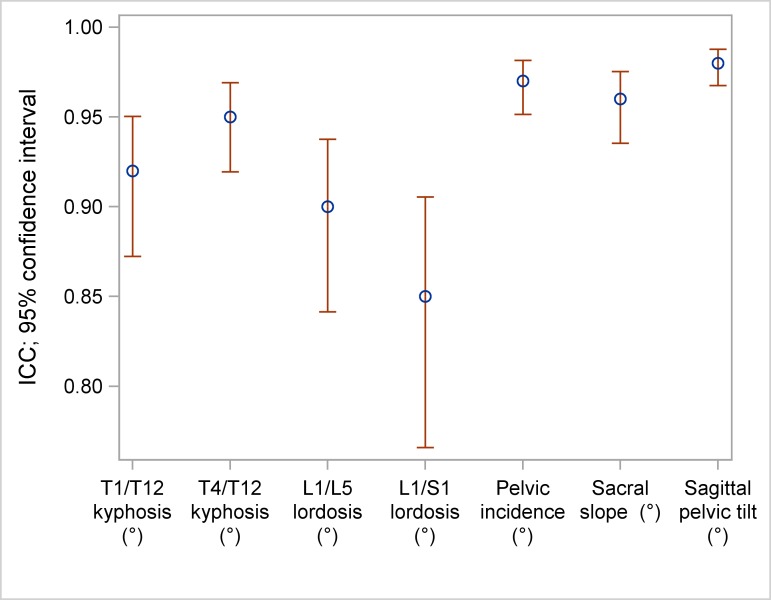
ICC and 95% confidence intervals (CI) of lordosis/kyphosis and pelvic parameters.

ICC was 0.92 for T1/T12 kyphosis, 0.95 for T4/T12 kyphosis, 0.9 for L1/L5 lordosis and 0.85 for L1/S1 lordosis. Concerning the pelvic parameters ICC was 0.97 for pelvic incidence, 0.96 for sacral slope, 0.98 for sagittal pelvic tilt and 0.94 for lateral pelvic tilt (Table 6, Fig 6).

**Table 6:** ICC and 95% confidence intervals (CI) of lordosis/kyphosis and pelvic parameters.

**Lordosis/kyphosis**	**ICC**	**95% CI**
**T1/T12 kyphosis**	0.92	[0.87; 0.95]
**T4/T12 kyphosis**	0.95	[0.92; 0.97]
**L1/L5 lordosis**	0.90	[0.84; 0.94]
**L1/S1 lordosis**	0.85	[0.77; 0.91]
**Pelvic parameters**	**ICC**	**95% CI**
**Pelvic incidence**	0.97	[0.95; 0.98]
**Sacral slope**	0.96	[0.94; 0.98]
**Sagittal pelvic tilt**	0.98	[0.97; 0.99]
**Lateral pelvic tilt**	0.94	[0.90; 0.96]

The ICC as well as the 95% confidence intervals were assessed for the lordosis/kyphosis measurements and for the pelvic parameters.

## Discussion

The limitations of 2D measurements based on two-plane X-rays and the advantages of measurements obtained from 3D reconstructions have been emphasized in former studies [14–16]. Apart from the Cobb-angle, the vertebral rotation is an important clinical parameter in the follow-up and the preoperative planning in patients with AIS. One disadvantage of CT which is able to provide accurate 3D measurements is its higher radiation exposure. The results of the radiation exposure analysis in this study show that the absorbed dose is 26 times lower with EOS compared to a full-spine CT and about 8 times lower than full-spine low-dose CT (Table 7).

**Table 7:** Mean radiation exposure of EOS compared to other techniques [16].

Technique	Full spine frontal	Full spine lateral
EOS	0.253	0.339
Conventional radiograph (CR)	1.662	1.862
CT-scan	15.6	
Low-dose CT-scan	5	

Radiation exposure (in mGy) of EOS was compared to the most common imaging techniques in spinal examination in case of scoliosis [17].

Abul-Kasim et al. described a method to assess vertebral rotation with a low dose CT-protocol with effective dose 20 times lower than a standard CT [18]. Although being able to reduce dose in CT, the main disadvantage of modified vertebral posture and alignment caused by the prone position in which the patients had to be examined, was still not solved. Compared to the EOS-system, the patients need to lie during the CT-scan leading to changes in static global balance.

With EOS, the time consuming compounding of X-rays acquired from conventional radiographs is not needed any more and radiation exposure is reduced up to 6 times compared to conventional radiograph (17). Presciutti et al. showed that the mean number of radiographs per year performed in patients with AIS is 3.5 in the observation group, 5.7 in the bracing group and 12.2 in the operated group [19]. Considering these data EOS is capable to save up to 36mGy absorbed dose per year which is, especially in children who need long-term follow-up, a considerable benefit with regard to the estimated life-time risk of developing radiation related cancer [17]. The 9.5 second scan-time of EOS full-spine examinations make it applicable in the routine clinical setting to all patients who are able to stand in an upright position. Even in obese patients acquisition time was not longer than 21 seconds which was still acceptable in most cases.

Other studies focusing on several spine and pelvic parameters (AVR, T1/T12 kyphosis, T4/T12 kyphosis, L1/L5 lordosis, L1/S1 lordosis, pelvic incidence, sacral slope, pelvic tilt and pelvic angle) confirmed the reproducibility of EOS 3D reconstructions [7,9,13,20].

However, a differentiated analysis on reliability of the three-dimensional rotation measurements of every single thoracolumbar vertebral body was still lacking. Here we show that interreader reproducibility of every single vertebra rotation from T1-L5 is very good to good for frontal and very good for lateral rotation measurement. Assessing the axial vertebral rotation in the lower thoracic and the lumbar spine was easy to perform with a good interreader reproducibility. In the upper and middle thoracic spine (T1-T7) the assessment of the axial vertebral rotation had some limitations resulting in a moderate ICC. The axial rotation results mainly from the adjustment of the pedicles and the posterior arch in the frontal view. Identifying these structures can be difficult in the upper and middle thoracic spine because of the lower diameter and the obtuse angle of the pedicles and the posterior arch in comparison to the lumbar spine.

In patients with scoliosis, lordosis and kyphosis are difficult to measure on lateral views of 2D CR compared to patients with no scoliosis because of the vertebral rotation and angle of the endplates with regard to the horizontal plane [21]. The interreader agreement was good to very good in matters of kyphosis/lordosis and very good for pelvic parameters using EOS and the “full spine” protocol in the sterEOS software which is in accordance with the results of former studies [7,9,13,20].

The mean time for reconstruction was 14.9 minutes (reader 1: 14.6 minutes, reader 2: 15.2 minutes). Former studies reported slightly lower mean reconstruction times ranging from 11.5 to 13.5 minutes which may be due to the fact that our focus in the reconstruction procedure was on the rotation of ever single vertebra which is time consuming as it needs dedicated fine tuning of the vertebral contour [7,9].

There are some limitations that need to be discussed. First, patients included in this study had a mild to moderate scoliosis with a mean Cobb angle of 18.2° (range, 9.8°–49.9°). According to the EOS recommendations, EOS 3D-modeling is limited in patients with a severe scoliosis (Cobb angle > 50°) because of the poor distinguishability of anatomical landmarks. Nevertheless, a follow-up study could investigate a cohort of patients with severe scoliosis regarding 3D-modeling and inter-reader reproducibility to expand the applicability of this modality. Second, 6 patients were excluded from the study because of lumbosacral transitional vertebrae and vertebral deformity. According to EOS guidelines the sterEOS software is not applicable in these patients because reconstructions and measurements would lead to false results. Third, 2 patients dropped out because of motion artifacts leading to blurred images. The scan-time of these two patients was 20.6 and 22.7 seconds, respectively (mean scan-time 9.5 seconds). Despite the many advantages EOS offers compared to digital radiography (DR) it is prone to motion artifacts with prolonged examination time. The scan-time depends on the physical constitution and is longer in obese patients. Patients who are not able to stand still for the duration of the examination are not suitable for an examination with EOS and should be examined with other modalities like DR or CT.

In conclusion 3D angle measurement of frontal, lateral and axial vertebral rotation proved to be reliable with very good and good results. Interreader reproducibility of axial rotation was limited to some degree in the upper and middle thoracic spine due to limited identifiability of the pedicles and the processi spinosi. Additionally, our results confirm the reliability of 3D measurements regarding pelvic parameters as well as kyphosis and lordosis measurements with sterEOS.
